# Cortisol and cognitive function in midlife: The role of childhood cognition and educational attainment

**DOI:** 10.1016/j.psyneuen.2014.05.018

**Published:** 2014-09

**Authors:** Darya Gaysina, Michael P. Gardner, Marcus Richards, Yoav Ben-Shlomo

**Affiliations:** a School of Psychology, University of Sussex, Brighton, UK; b School of Social and Community Medicine, University of Bristol, Bristol, UK; c MRC Unit for Lifelong Health and Ageing, University College London, UK

**Keywords:** Cognition, Development, Stress, Cortisol, Memory, Ageing

## Abstract

Adult cognition and age-related cognitive decline can be influenced by dysregulation of the hypothalamic pituitary adrenal axis with concomitant changes in cortisol levels. However, very little is known about the role of childhood cognition and educational attainment in this relationship. Using data from the British 1946 birth cohort, the present study investigated: (1) associations between cortisol levels and patterns and cognitive function in midlife; (2) direct and interactive effects of childhood cognition, educational attainment and cortisol on cognitive function in midlife. Verbal memory, letter search speed and reaction time were assessed at age 60–64 years. Salivary cortisol samples (wakening, 30 min after wakening and evening) were collected at the same age. Childhood cognitive ability was measured at ages 8, 11, and 15, and educational level was reported at age 26. Associations between cortisol, childhood cognition, educational attainment and cognitive function in midlife were tested using linear regression and structural equation modelling approaches. Higher evening cortisol level was associated with slower reaction time and lower verbal memory. These associations were independent of childhood cognition and education as well as a range of other potential confounders. Childhood cognition and education were not directly associated with evening cortisol. However, there was a significant interaction effect between childhood cognition and evening cortisol on reaction time (*p* = .002): higher evening cortisol was associated with slower reaction time only among those with low childhood cognitive ability. There was little evidence of associations between the other cortisol measures and cognitive function.

Adult cognition and age-related cognitive decline can be influenced by dysregulation of the hypothalamic pituitary adrenal axis (HPA-axis), which produces cortisol. Cortisol is known to regulate various brain functions (Belanoff et al., 2001), with well-described effects on human cognition (Davis, 1986; Lupien et al., 2005). In some studies, chronic exposure to high cortisol levels (e.g., high morning or evening cortisol, higher mean over the day and flatter diurnal drop) have been associated with poorer cognitive functioning, accelerated cognitive ageing and Alzheimer's disease (Kalmijn et al., 1998; Karlamangla et al., 2005; Lupien et al., 2005; Li et al., 2006; Kuningas et al., 2007; Beluche et al., 2010; Rothman and Mattson, 2010). For example, Beluche et al. (2010) demonstrated that high morning salivary cortisol levels were associated with a decline in visual memory in women, and a flatter diurnal slope was associated with a decline (over 4 years) in cognitive function in both men and women aged 65–90 years. In a study by Karlamangla et al. (2005), overnight urinary cortisol levels were associated with cognitive decline over a 7 year follow-up in a sample of women and men aged 70–79 years. In a study by Li et al. (2006), higher night time salivary cortisol was associated with a decline in paragraph recall, although this small study was restricted to 46 participants at follow-up (mean age 78 ± 7 years). However, a study by Comijs et al. (2010), found no association between morning serum cortisol and cognitive decline over a period of 6 years. The inconsistency in the results of the above mentioned studies might be explained by their methodological differences; for example, cortisol was measured in a variety of ways including in saliva (Beluche et al., 2010), serum (Comijs et al., 2010) or urine (Karlamangla et al., 2005). Moreover, outcome measures of cognitive performance are also heterogeneous in the literature (for more detail, see review (Ben-Shlomo et al., 2013).

Cortisol typically follows a marked diurnal rhythm, with high levels in the morning that peak 30–45 min after waking, dropping rapidly for the next several hours and declining slowly throughout the rest of the day, until at a low point of around midnight (Adam et al., 2006). Overall, considerable variation in the diurnal pattern exists across individuals, as well as within individuals (Hruschka et al., 2005). In modelling cortisol as an outcome, different features of the diurnal pattern need to be examined (e.g. slope of the diurnal curve from peak to trough, the size of the cortisol awakening response (CAR), levels of morning and/or evening cortisol levels, and measures of free cortisol release over the day such as area-under-the-curve). We have previously postulated that HPA dysregulation may follow a natural history so that initially hyper-responsiveness (heightened CAR) may, with greater chronicity, evolve into hyporesponsiveness; attenuated CAR and smaller diurnal variability over the day (Ben-Shlomo et al., 2013). Several previous studies have investigated the link between cortisol patterns and cognitive functioning with diurnal decline measures (e.g. O’Hara et al., 2007; Beluche et al., 2010), however, other studies with repeat cortisol measures over the day have reported the area under the curve (AUC) as the strongest predictor of worse cognition (e.g. Lee et al., 2007; Franz et al., 2011). Many of the existing studies used small unrepresentative samples. Therefore, it is necessary to explore association between cortisol and cognitive function in midlife in a large population-representative sample utilising measures of cortisol that allow modelling of diurnal cortisol patterning.

Moreover, very little is known about the role of childhood cognition and educational attainment in this relationship. Three possible mechanisms of these early influences on midlife cognitive function can be proposed: (1) effects of cortisol on adult cognition are independent of childhood cognition and educational attainment; (2) childhood cognition and/or educational attainment can confound the association between cortisol and midlife cognitive function; (3) there might be an interaction effect between childhood cognition and/or educational attainment and cortisol on adult cognition (Potvin et al., 2013).

The present study investigated: (1) whether there were associations between cortisol levels over the day and patterns and cognitive function in midlife, and (2) the role of childhood cognition and education as potential effect modifiers using data from the National Survey of Health and Development (NSHD), a prospective population-based study. A particular advantage of this cohort was the availability of cognition measured in childhood, which was previously shown to be inversely associated with morning cortisol level and waking response in the British 1958 birth cohort (Power et al., 2008), and was also associated with midlife and later life cognitive function (Deary et al., 2000; Richards et al., 2003; Richards and Sacker, 2003; Gow et al., 2012).

## 1 Method

### 1.1 Sample

The NSHD (also known as the British 1946 birth cohort) is a socially stratified birth cohort, originally consisting of 2547 women and 2815 men, who have been followed up since their birth in 1946 with regular data collections, most recently at age 60–64 (henceforth 60+) years (Kuh et al., 2011). At this data collection, contact was sought with 3163 study members still alive and living in Great Britain with a known address. The majority (*n* = 2662, 84% of the target sample) provided information, and 2229 were interviewed and examined in the clinics (*n* = 1690) or if preferred in their own homes (*n* = 539) by trained research nurses. Those interviewed at age 60+ were, in most respects, representative of the national population of that age apart of lower educational attainment, lower childhood cognition and lifelong smoking (Stafford et al., 2013). No attempt was made to contact the remaining 2199 study members: 718 (13.4%) had already died, 594 (11.1%) had previously withdrawn from the study, 567 (10.6%) lived abroad and 320 (5.9%) had been untraceable for more than ten years. Ethical approval for this research was obtained from the North Thames Multi-Centre Research Ethics Committee, and from relevant local research ethics committees in the survey areas. Informed consent was given by all study members.

## 2 Measures

### 2.1 Midlife cognitive function

Three cognitive tests were administered at age 60+ years: (1) a verbal memory task requiring to remember words from 15 word list, with three learning trials and free recall at the end of each; (2) a timed visual search task requiring cancellation of target letters P and W, embedded among non-target letters (mean of three trials); and (3) simple reaction time, requiring survey members to press a button as quickly as possible following a signal, with eight practice trials being given, followed by 20 real trials. All test scores were standardised to give a mean of 0 and a standard deviation of 1 (*z*-score), based on the sample with non-missing values for each variable used in this study.

### 2.2 Salivary cortisol measures

NSHD study members were requested to collect saliva samples: at awakening (t1), 30 min after waking (t2), and between 2100 h–21.30 h the same evening (t3). A mid-morning sample was also taken at the clinic visit but has not been used in this study. Participants were instructed to avoid brushing or flossing their teeth, or eating, drinking or (if applicable) smoking for 30 min before taking each sample. They were asked to place a Salivette saliva swab in their mouth until it was soaked, record the date and time of collection, and store the sample in the refrigerator (but not in the freezer compartment) until posted to the laboratory in a pre-paid and protected container. Salivary cortisol is stable at room temperature for up to 30 days but samples were frozen after reaching the laboratory to reduce microbial growth.

Samples were subsequently assayed by radioimmunoassay in the laboratory specialising in high through-put cortisol assays (Dresden; Kirschbaum and Hellhammer, 1989).

The cortisol collection was not initially included in the protocol and so 348 study members living in the Manchester area of the UK, who constituted the feasibility sample at this sweep, were not invited to take part in this element of the study. Samples on at least one cortisol measure were received from 1796 participants (95.5% of the 1881 participants asked). Participants also recorded: (i) the precise time they took each sample; (ii) whether they ate and/or drank 30 min before the samples taken; (iii) whether they suffered stress, anxiety or trauma during the hour before the samples taken; and (iv) whether they smoked 30 min before the samples taken. There were a few positive responses which were omitted from the analyses.

The resulting cortisol data were examined in five ways. First, the three individual samples, wakening (t1), 30 min after wakening (t2) and evening cortisol (t3) were considered, since it has been proposed that these may be more sensitive indicator of HPA axis activity (Powell et al., 2002; Grossi et al., 2005). Two further variables – cortisol awakening response (CAR) (t2–t1) and diurnal drop (DD) (t3–t1) were derived to maximise the comparability of our measures with previous research. These are measures commonly used in epidemiological studies (Adam and Kumari, 2009). CAR was measured as the difference between the 30 min post waking sample and the waking sample. Since there is no standard way to measure DD, this variable was obtained by subtracting the evening sample from the morning sample. We also run a sensitivity analysis for this variable by using the average of the two morning samples ((t1 + t2)/2) to reduce any measurement error in the morning sample (Gardner et al., 2011).

### 2.3 Potential confounders

#### 2.3.1 Childhood cognitive ability

A growing body of evidence suggests that childhood cognition may protect against cognitive decline in midlife and beyond (for example, Richards et al., 2004). Moreover, it has been demonstrated that childhood cognition can influence cortisol levels and patterns (Power et al., 2008).

NSHD children were tested by teachers in a school setting at ages 8, 11 and 15 years using tests of verbal and nonverbal ability devised by the National Foundation for Educational Research. At age 8 these tests were: (1) reading comprehension (selecting appropriate words to complete 35 sentences); (2) word reading (ability to read and pronounce 50 words); (3) vocabulary (ability to explain the meaning of 50 words); and (4) picture intelligence, consisting of a 60-item non-verbal reasoning test. At age 11 the tests administrated were: (1) word reading (ability to read and pronounce 50 words); (2) vocabulary (ability to explain the meaning of 50 words); (3) Alice Heim Group Ability Test (AH4), a 80 item timed test, with separate verbal and non-verbal sections. The verbal items consist of analogies, comprehension, and numerical reasoning, while the non-verbal items consist of matching, spatial analysis, and non-verbal reasoning; and (4) arithmetic test (50 tasks). At age 15 these tests were: (1) Alice Heim Group Ability Test (AH4), a 130 item timed test, with separate verbal and non-verbal sections; (2) The Watts-Vernon reading test, a test of reading comprehension requiring the participant to select appropriate words to complete 35 sentences; (3) A 47 item mathematics test, requiring the use of arithmetic, geometry, trigonometry, and algebra. For each age, a summary variable of cognitive ability was derived using the average score across the tests administered at this age. Then, a summary variable of childhood cognition was derived using the average scores of the three ages (8, 11 and 15) if available. If the data were only available for the two ages, the summary variable was derived using the average of two.

#### 2.3.2 Educational attainment

Higher educational attainment is associated with better cognitive function in adulthood (Richards and Sacker, 2003; Hatch et al., 2007; Clouston et al., 2012) and can influence cortisol levels and patterns (Brandtstadter et al., 1991; Cohen et al., 2006; Dowd et al., 2011).

The highest educational or training qualification achieved by survey members by age 26 years was classified by the Burnham scale (Department of Education and Science, 1972). From this scale they were grouped into no qualification, below ordinary secondary qualifications (vocational), ordinary secondary qualifications (‘O’ levels and their training equivalents), advanced secondary qualifications (‘A’ levels and their equivalents), or higher qualifications (degree or equivalent).

Other potential confounders were sex, age at testing, socioeconomic position (SEP), lifetime smoking status, affective symptoms and adult BMI. Adult SEP was defined by own occupation, classified as manual (skilled manual, semi-skilled manual, and unskilled) and non-manual (professional, managerial or intermediate). Lifetime smoking status (lifelong smoker, predominantly smoker, predominantly non-smoker, never smoker) was based on reports of smoking behaviour at all contacts since age 20 years (Clennell et al., 2008). BMI (weight kg/height m^2^) was used as a marker of adult adiposity. Affective symptoms at age 60+ were represented by the total score of the General Health Questionnaire (Goldberg and Hillier, 1979). The GHQ is a screening device for identifying minor (non-psychotic) psychiatric disorders in the general population; the threshold of score of 5 or more can be used to define ‘caseness’. By applying this threshold, the total of 16.8% survey members included in the current analysis have met the criteria for common mental health problems. All of these variables are known to have effects on adult cognitive and HPA-axis functions.

### 2.4 Analytical procedure

#### 2.4.1 Regression analyses

First, we tested for associations between the five cortisol variables (i.e., wakening, 30 min after wakening, evening, CAR, and DD) and cognitive function at age 60+ using linear regression models. For all analyses, cortisol levels were regressed on time of sample collection indicated on the self-completed form, and residuals were then used in the analysis; this was to control for any noise caused by differences between study members in precise time of sample collection. Night time cortisol was positively skewed and we therefore transformed it (log_e_). All cortisol measures were standardised to give a mean of 0 and a standard deviation of 1.

The initial analysis was done in the sample with the maximum number of participants. Sex by cortisol measures interaction terms were fitted to test for effect modification by sex. The following steps were performed in the sample with the complete information on all covariates. First, the model was fitted controlling for sex and age at testing only. Second, the covariates of SEP, lifetime smoking status, affective symptoms and BMI were added into the model. Then childhood cognition (average for cognitive tests scores at ages 8, 11 and 15) was added. Finally, educational attainment was added. In addition, we tested for the interaction effects between cortisol and childhood cognition, and between cortisol and educational attainment on adult cognition.

#### 2.4.2 Structural equation modelling

Following results from the linear regression analyses, we examined associations between childhood cognition, educational attainment, and cortisol on cognitive function in midlife using SEM. We only estimated SEMs where cortisol-cognition associations were significant in the regression models. Model estimation was performed with Stata 12 (StataCorp. 2011). The fit of the models were evaluated with recommended fit indices including the Tucker–Lewis index (TLI), the root mean square error of approximation (RMSEA), and the comparative fit index (CFI). Models are generally considered as having ‘good fit’ if they achieve > 0.95 on the CFI and TFI, and RMSEA value below 0.06. The structural part of the SEM model represents the latent variable of childhood cognition constructed with three indicators of cognition at ages 8, 11 and 15. The measurement part of the SEM model tests for the relationship between childhood cognition, educational attainment, cortisol levels and cognitive function at age 60+.

## 3 Results

### 3.1 Descriptive analysis

Descriptive statistics for adult cognitive function (i.e., three cognitive tests scores), cortisol measures, childhood cognition, and educational attainment are presented in Table 1, for males and females separately. Women had better cognitive performance for verbal memory (*p* < .001) and letter search speed (*p* = .003) at 60+ but had slightly worse cognition at age 15 (*p* < .001). Educational levels were lower for women compared to men (*p* < .001). Women had a lower wakening level of cortisol (*p* < .001) and greater CAR (*p* = .001) but very similar diurnal drop. Since no sex interactions for cortisol measures were observed (*p* > .10), all further analyses were performed in the combined group adjusting for sex.

Study members with at least one cortisol measure (*n* = 1796) were included in the analysis. Those who did not provide cortisol samples during the latest data collection (*n* = 434) were more likely to be male (*p* = .04), had lower childhood cognitive ability (*p* = .02), and slower reaction time (*p* = .001) but did not have significantly different levels of education (*p* = .09), verbal memory (*p* = .22) or letter search speed (*p* = .55).

### 3.2 Regression analysis

The only association between cortisol levels and the cognitive tasks was for the night time sample which showed that higher cortisol levels were associated with worse verbal memory (*p* = .002) and slower reaction time (*p* = .002) but chance associations with letter search speed (*p* = .09) (Table 2). Sensitivity analysis with the modified variable of diurnal drop produced the results similar to the ones from the main analysis shown at Table 2 (data available on request).

Modelling associations between evening cortisol level and verbal memory in the sample with complete information on all covariates (*n* = 1030) found a similar result (*β* = −0.072, SE = 0.030, *p* = .02; Table 3, Model 1). This association remained statistically significant after adjustment for SEP, affective symptoms, lifetime smoking status and BMI (*β* = −0.06, SE = 0.03, *p* = .03; data available upon request); this effect remained statistically significant after additional adjustment for childhood cognition (*β* = −0.06, SE = 0.025, *p* = .02; data available upon request), and after additional adjustment for educational attainment (*β* = −0.057, SE = 0.025, *p* = .02; Table 3, Model 2). As Table 3 shows, there were statistically significant effects of childhood cognition (*β* = 0.363, SE = 0.036, *p* < .001) and educational attainment (*β* = 0.131, SE = 0.024, *p* < .001) on verbal memory score.

Modelling associations between evening cortisol level and letter search speed in the sample with complete information on all covariates (*n* = 1093) found a similar borderline significant result (*β* = −0.063, SE = 0.03, *p* = .04; Table 4, Model 1). This association was slightly attenuated after adjustment for all covariates and was not longer statistically significant (*β* = −0.058, SE = 0.03, *p* = .05; Table 4, Model 2).

The association between evening cortisol level and reaction time was confirmed in the sample with complete information on all covariates (*n* = 1030, *β* = 0.091, SE = 0.030, *p* = .002, Table 5, Model 1). This association remained statistically significant after adjustment for SEP, affective status, life course smoking status and adult BMI (*β* = 0.086, SE = 0.029, *p* = .004; data available upon request); this association remained statistically significant after additional adjustment for childhood cognition (*β* = 0.085, SE = 0.029, *p* = .004; data available upon request) and educational attainment (*β* = 0.085, SE = 0.029, *p* = .004; Table 5, Model 2). As Table 3 shows, there was a significant effect of childhood cognition (*β* = −0.168, SE = 0.036, *p* < .001) and but not of educational attainment (*β* = −0.008, SE = 0.028, *p* = .79) on reaction time.

**Figure 1:**
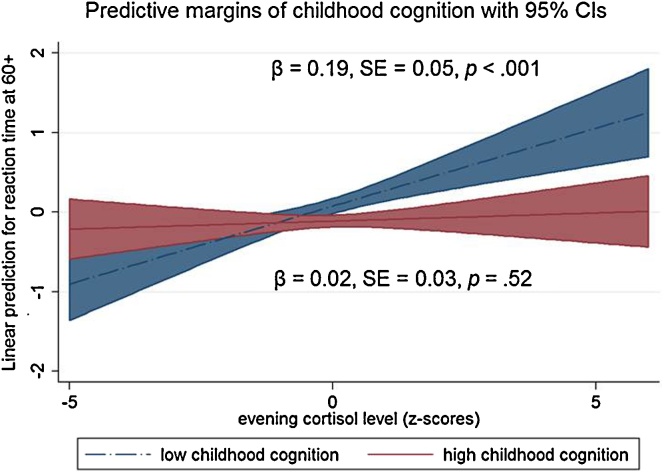
Linear prediction of reaction time score at age 60–64 by evening cortisol level and childhood cognitive ability; *p*-value for interaction = .002 (based on the continuous measure of childhood cognition).

We have also tested for interaction effects of childhood cognition and cortisol measures on adult cognition. There was a significant interaction effect between childhood cognitive ability and evening cortisol level on reaction time at age 60+ (*p* for interaction = .002). In order to clarify the interaction effect, a binary variable of childhood cognitive ability (high versus low) was derived using the mean of the summary score as a threshold. Stratified analysis using this binary variable of childhood cognitive ability revealed that there was a significant association between evening cortisol level and reaction time in the group with low childhood cognition (*β* = 0.19, SE = 0.05, *p* < .001), but not in the group with high childhood cognition (*β* = 0.02, SE = 0.03, *p* = .52; Fig. 1). A similar interaction pattern was observed with childhood cognition and midlife reaction time on evening cortisol (*p* = .04): only in those with low childhood cognition, association between reaction time and nighttime cortisol was observed (*β* = 0.16, SE = 0.05, *p* < .001).

There were no interaction effects between childhood cognition and other cortisol measures, on verbal memory, letter search speed and reaction time. Similarly, no interaction effects were observed for educational attainment and cortisol on midlife cognition (data available on request).

Since associations between childhood cognition and morning cortisol and CAR in midlife have previously been reported in the British 1958 birth cohort, we attempted to replicate these findings. There were no significant associations between a continuous measure of childhood cognition and morning cortisol (*p* = .36) or CAR (*p* = .24). Those within the lowest quartile of childhood cognition did not differ from those within the highest quartile in relation to morning cortisol level (*p* = .45) or CAR (*p* = .38).

### 3.3 SEM analysis

**Figure 2:**
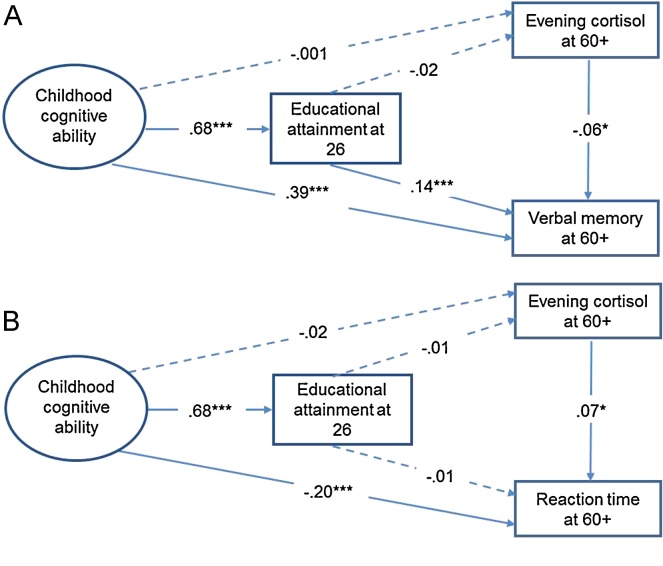
Results of the SEM showing the effects of childhood cognition, educational attainment and evening cortisol on cognitive function at age 60–64: (A) verbal memory; (B) reaction time; models are adjusted for sex, age at testing, adult socio-economic position, life-course smoking status, BMI and affective status; standardised coefficients shown; *p < .05, ***p < .001.

The results of the SEM models are presented in Fig. 2A and B. Higher evening cortisol level was associated with lower verbal memory at age 60+ (*B* (unstandardised coefficient) = −0.05, *SE B* = 0.03, *β* (standardised coefficient) = −0.06, *p* = .04). As expected, there was a direct effect of childhood cognitive ability on verbal memory (*B* = 0.49, *SE B* = 0.05, *β* = 0.39, *p* < .001) and childhood cognitive ability also significantly predicted educational attainment at age 26 (*B* = 1.25, *SE B* = 0.06, *β* = 0.68, *p* < .001), which in turn significantly predicted verbal memory at age 60+ (*B* = 0.09, *SE B* = 0.03, *β* = 0.14, *p* = .001). There were no significant direct effects of childhood cognitive ability or educational attainment on evening cortisol level (Fig. 2A).

Higher evening cortisol level was also associated with slower reaction time at age 60+ (*B* = 5.84, *SE B* = 1.9, *β* = 0.07, *p* = .003). There was a direct effect of childhood cognition on reaction time (*B* = −11.55, *SE B* = 2.92, *β* = 0.20, *p* < .001), but not on evening cortisol level in midlife. There were no significant associations between educational attainment at age 26 and reaction time or cortisol level (Fig. 2B).

## 4 Discussion

The present study utilised a longitudinal cohort design to examine the cross-sectional association between cortisol levels and patterns on cognitive function in midlife, and to investigate the role of prospectively measured childhood cognitive ability and educational attainment in this relationship. Both the linear regression and SEM results indicated a significant association between higher evening cortisol level and lower verbal memory and slower reaction time at age 60+. These associations were not confounded by childhood cognition or educational attainment, suggesting that the effects of evening cortisol on cognitive performance in midlife were independent from the effects of these early life influences. Moreover, the effect of evening cortisol on reaction time varied depending on childhood cognitive ability; the effect was significantly stronger for those with lower cognitive ability in childhood. This observed interaction effect suggests that cognitive function in midlife is more likely to be associated with cortisol among those with lower childhood cognitive ability.

Our findings are somewhat in agreement with some of the previous studies showing the association between higher cortisol levels and worse cognitive performance in midlife (Karlamangla et al., 2005; Lupien et al., 2005; Li et al., 2006; O’Hara et al., 2007; Beluche et al., 2010; Geoffroy et al., 2011). In a longitudinal study of 538 men and women, 70–79 years of age, higher overnight urinary cortisol at baseline was associated with higher risk of incident cognitive impairment over the 7-year follow up (Karlamangla et al., 2005). However, most of the existing studies did not investigate the effect of evening cortisol on cognitive function, but rather focused on morning cortisol, diurnal drop or AUC. For example, a recent study by Geoffroy et al. (2011) using longitudinal data reported that higher cortisol levels in late morning at age 45 were associated with poorer verbal memory and fluency at age 50. In this study, only two measures of cortisol, 45 min after waking (t1) and 3 h later (t2), were available, therefore this study was not able to explore the effect of evening cortisol level or disruption of the normal diurnal rhythm of cortisol that might be important for cognition.

The normal diurnal rhythm is characterised by a post-waking peak and subsequent decline over the day, but with approximately 10% of individuals lacking the post-waking peak (Stone et al., 2001). It has been argued that a lack of an early morning cortisol peak could reduce a person's capacity to remain alert during the day (Gunnar and Vazquez, 2001). Alternatively, this may reflect measurement error as studies show that subjects with worse compliance do not take the first cortisol sample immediately on waking so that the CAR appears artefactually attenuated and this is more likely to occur in subjects with lower educational level (Golden et al., 2014). In the present study, we did not show any association between childhood cognition and waking cortisol or CAR in midlife. Thus, we failed to confirm the findings from the study by Power et al. (2008) of the association between childhood cognition and cortisol awakening response in midlife. We did not show any concurrent association between morning cortisol or CAR and cognitive function in midlife, but rather we demonstrated that higher evening cortisol was linked to worse performance on three cognitive tasks – verbal memory, letter search speed and reaction time.

Several investigators have suggested that high cortisol levels impair memory consolidation in midlife (Herbert et al., 2006). Higher cortisol levels are thought to lead to suppression as opposed to potentiating under-stimulation of hippocampal mediated learning and memory (Belanoff et al., 2001; Lupien et al., 2005; Haley et al., 2006).

Interestingly, the effect of evening cortisol on reaction time was modified by childhood cognition. Cognitive function in midlife was more likely to be affected by cortisol if childhood cognitive ability was lower. This observation is consistent with the hypothesis that childhood cognitive ability may acts as a resilience factor, such that those with higher levels of cortisol would be less likely to have cognitive dysfunction in later life if they had better childhood cognitive ability. This observation is consistent with evidence that higher intelligence and educational attainment may exert a protective effect in the presence of chronic psychosocial stress (Rutter, 1979).

The mechanism through which childhood IQ modify the effect of cortisol on cognitive performance in midlife is unknown. There are several possible explanations. First, lower childhood IQ may be a marker of neuroanatomical problems that increase vulnerability to cognitive dysfunction in later life. For example, IQ is positively correlated with cerebellar volume (McDaniel, 2005), and verbal IQ has been shown to correlate with hippocampal volume in male children (Schumann et al., 2007). Exposure to high cortisol level has been associated with smaller hippocampal volumes in patients with Cushing's syndrome (Starkman et al., 1992) and in the normal elderly (Lupien et al., 1998). Second, individuals with lower childhood cognition may be less equipped to cope with stressful life events, making them potentially more vulnerable to developing cognitive dysfunction after exposure to stress.

In a study by Potvin et al. (2013), the modifying effect of education on the association between cortisol and cognition in an elderly sample (aged 65 and above) was reported: in high educated participants, but not in low educated participants, high morning cortisol level was associated with prevalent cognitive impairment and high afternoon cortisol level increased the risk of incident cognitive impairment. In our study, we did not observe a significant direct effect of morning cortisol, or an interaction effect with education, on midlife cognition. Future studies are needed to follow-up the initial findings of modifying effects of childhood cognition and education on the associations between cortisol and cognition in midlife.

### 4.1 Study limitations

Whereas the ability to examine the role of childhood cognition and educational attainment on the association between cortisol level and cognitive function in midlife based on the data from the national population-based sample is a primary strength of the present study, the observed association between higher evening cortisol and cognitive function in midlife was cross-sectional. This limits the ability to draw inference between these constructs, which would be strengthened by longitudinal separation (Rutter, 2007). In order to clarify the direction of the effect, information on cortisol patterns in early life, followed by data on cognitive trajectories and cortisol outcome some decades later would be required.

Despite this limitation, the prospective measures of childhood cognition and education were available in the present study. We demonstrated that cognitive ability in childhood did not explain this association in midlife. In line with this observation, the Vietnam Era Twin Study of Aging showed that a cross-sectional association between salivary cortisol and cognitive functioning at age 51–55 years were not explained by cognitive ability at age 20 (Franz et al., 2011). Moreover, the direction of effects observed in the present study fits with the pattern of effects observed in past research (Geoffroy et al., 2011) allowing for a more confident assumption that cortisol level might be a risk factor for cognitive dysfunction in midlife.

An additional limitation of the present study is that the observed association might be driven by a confounder. We controlled for a number of health and lifestyle factors, such as adult SEP, lifetime smoking status, affective symptoms and BMI. We showed that these factors only partially confounded the association between higher evening cortisol and cognitive ability in midlife. However, some unmeasured aspects may still account for this relationship.

Diurnal cortisol was captured over a single 24 h period though it varies from day to day. Therefore, there might be some measurement error of the available cortisol data in characterising the HPA axis. Indeed, it has been recommended that CAR to be performed at least twice on two separate days (Hellhammer et al., 2007).

Finally, losses to follow-up and missing data are unavoidable in long running birth cohort studies such as the NSHD. For example, those with lower childhood cognitive ability were under-represented in this study. However, the maximum likelihood estimator was applied in the SEM and a missing at random assumption was applied to the missing cases. Moreover, there is no reason to suspect that any differences in the characteristics of those with missing data compared with the rest would have a substantial impact on our finding of association between midlife evening cortisol and cognitive function.

Notwithstanding these limitations, the present study adds to the literature on the relationship between cortisol levels and cognitive function in midlife, while also considering the relative role of childhood cognition and educational attainment, in a national birth cohort sample. In terms of future directions, and given estimates of the acceleration of global population ageing (Lutz et al., 2008), it is important to explore factors and processes across the lifespan that underlie risk and resilience to cognitive dysfunction in later life. While evidence supports the conclusion that prolonged exposure to stress can affect cognitive function, very little is known about the role of factors that can modify this relationship. The present study is one of the first to explore the potential role of childhood cognition and educational attainment as factors that can modify the negative effects of cortisol on cognitive function in midlife. Yet examination of the neurobiological mechanisms that might underlie this resilience represents an important area of future research.

## Role of the funding source

The NSHD is supported by the Medical Research Council. The funders did not play any role in study design, data analysis, decision to publish, or preparation of the article.

## Conflict of interests

The authors have no conflict of interest to declare.

## Figures and Tables

**Table 1:** Descriptives for midlife cognitive performance, cortisol measures and childhood cognition and educational attainment in the British 1946 birth cohort; mean (SD) shown unless specified.

Variables	Males	Females	*p*-Value
Cognitive performance at 60–64:			
Verbal memory	−0.19 (0.96) *n* = 804	0.19 (0.99) *n* = 933	<.001
Letter search speed	−0.08 (0.99) *n* = 819	0.06 (0.99) *n* = 944	.003
Reaction time	0.00 (1.01) *n* = 814	−0.07 (0.88) *n* = 934	.12
Cortisol at 60–64 (nmol/L):			
Wakening	21.7 (11.4) *n* = 780	18.8 (9.2) *n* = 894	<.001
30 min after awakening	26.6 (11.9) *n* = 722	26.4 (11.1) *n* = 809	.68
Evening	3.42 (4.96) *n* = 691	3.38 (3.84) *n* = 780	.84
Cortisol awakening response	4.80 (11.9) *n* = 614	7.34 (11.6) *n* = 699	.001
Diurnal drop	23.9 (12.4) *n* = 547	23.9 (11.5) *n* = 608	.92
Childhood cognition:			
Cognitive ability at 8	0.11 (0.99) *n* = 745	0.17 (0.93) *n* = 847	.26
Cognitive ability at 11	0.17 (0.95) *n* = 733	0.20 (0.89) *n* = 817	.47
Cognitive ability at 15	0.29 (0.96) *n* = 712	0.14 (0.87) *n* = 810	.001
Overall childhood cognition	0.21 (0.96) *n* = 757	0.18 (0.88) *n* = 846	.64
Educational level at 26:			<.001
Degree or higher	18.2% *n* = 144	6.6% *n* = 60	
GCE ‘A’ level, Burnham B or A2	30.6% *n* = 242	27.0% *n* = 244	
GCE ‘O’ level or Burnham C	13.5% *n* = 107	26.6% *n* = 241	
Sub GCE or vocational course	6.3% *n* = 50	9.5% *n* = 86	
None	31.4% *n* = 248	30.3% *n* = 274	

**Table 2:** Associations between cortisol measures and cognitive function at age 60–64; models adjusted for sex and age at testing.

Cortisol measures	Cognitive performance at 60–64					
	Verbal memory		Letter search speed		Reaction time	
	*β* (SE)	*p*-Value	*β* (SE)	*p*-Value	*β* (SE)	*p*-Value
Waking	−0.002 (0.024)	.93	−0.015 (0.025)	.53	−0.003 (0.023)	.90
30 min after wakening	−0.022 (0.026)	.40	−0.040 (0.027)	.15	−0.003 (0.025)	.90
Evening	−0.08 (0.026)	.002	−0.045 (0.026)	.09	0.079 (0.025)	.002
Cortisol awakening response	−0.003 (0.026)	.90	−0.014 (0.028)	.61	−0.028 (0.025)	.26
Diurnal drop	−0.02 (0.027)	.45	−0.033 (0.029)	.27	−0.011 (0.027)	.68

**Table 3:** Results of linear regression models for verbal memory at age 60–64 (*n* = 1077).

	Model 1		Model 2	
	*β* (SE)	*p*-Value	*β* (SE)	*p*-Value
Evening cortisol	−0.072 (0.030)	.02	−0.057 (0.025)	.02
Sex	0.376 (0.058)	<.001	0.512 (0.050)	<.001
Age at testing	−0.016 (0.003)	<.001	−0.012 (0.002)	<.001
Adult SEP			−0.087 (0.023)	<.001
Life course smoking			0.000 (0.000)	.70
Adult BMI			−0.009 (0.017)	.57
Current affective status			−0.007 (0.003)	.01
Childhood cognition			0.363 (0.036)	<.001
Educational attainment			0.131 (0.024)	<.001

Model 1: adjusted for sex and age at testing; Model 2: fully adjusted for sex, age at testing, adult SEP, life-course smoking status, affective status, BMI, childhood cognition and educational attainment.

**Table 4:** Results of linear regression analyses for letter search speed at age 60–64 (*n* = 1093).

	Model 1		Model 2	
	*β* (SE)	*p*-Value	*β* (SE)	*p*-Value
Evening cortisol	−0.063 (0.030)	.04	−0.058 (0.030)	.05
Sex	0.136 (0.060)	.02	0.185 (0.060)	.002
Age at testing	−0.004 (0.003)	.27	−0.002 (0.003)	.48
Adult SEP			−0.027 (0.028)	.33
Life course smoking			−0.000 (0.000)	.83
Adult BMI			−0.004 (0.020)	.83
Current affective status			−0.002 (0.004)	.55
Childhood cognition			0.131 (0.044)	.003
Educational attainment			0.054 (0.029)	.06

Model 1: adjusted for sex and age at testing; Model 2: fully adjusted for sex, age at testing, adult SEP, life-course smoking status, affective status, BMI, childhood cognition and educational attainment.

**Table 5:** Results of linear regression analyses for reaction time at age 60–64 (*n* = 1082).

	Model 1		Model 2	
	*β* (SE)	*p*-Value	*β* (SE)	*p*-Value
Evening cortisol	0.091 (0.030)	.002	0.085 (0.029)	.004
Sex	−0.084 (0.058)	.15	−0.120 (0.059)	.04
Age at testing	0.008 (0.003)	.005	0.007 (0.003)	.02
Adult SEP			0.040 (0.027)	.14
Life course smoking			−0.000 (0.000)	.03
Adult BMI			−0.003 (0.020)	.95
Current affective status			0.003 (0.004)	.48
Childhood cognition			−0.168 (0.028)	<.001
Educational attainment			−0.008 (0.028)	.79

Model 1: adjusted for sex and age at testing; Model 2: fully adjusted for sex, age at testing, adult SEP, life-course smoking status, affective status, BMI, childhood cognition and educational attainment.
